# Women’s gambling behaviour, product preferences, and perceptions of product harm: differences by age and gambling risk status

**DOI:** 10.1186/s12954-018-0227-9

**Published:** 2018-04-24

**Authors:** Simone McCarthy, Samantha L. Thomas, Melanie Randle, Amy Bestman, Hannah Pitt, Sean Cowlishaw, Mike Daube

**Affiliations:** 1 Centre for Population Health Research, Faculty of Health, School of Health and Social Development, Deakin University, Geelong, Victoria Australia; 2 Faculty of Business, School of Management, Operations and Marketing, University of Wollongong, Wollongong, Australia; 3 Phoenix Australia Centre for Posttraumatic Mental Health, Department of Psychiatry, The University of Melbourne, Melbourne, Australia; 4 Bristol Medical School, University of Bristol, Bristol, UK; 5 Faculty of Health Sciences, Curtin University, Perth, Australia

**Keywords:** Women, Gambling, Sports betting, Electronic gambling machines, Public health

## Abstract

**Background:**

Women’s participation in, and harm from gambling, is steadily increasing. There has been very limited research to investigate how gambling behaviour, product preferences, and perceptions of gambling harm may vary across subgroups of women.

**Methods:**

This study surveyed a convenience sample of 509 women from Victoria and New South Wales, Australia. Women were asked a range of questions about their socio-demographic characteristics and gambling behaviour. Focusing on four gambling products in Australia—casino gambling, electronic gambling machines (EGMs), horse betting, and sports betting—women were asked about their frequency of participation, their product preferences, and perceptions of product harms. The sample was segmented a priori according to age and gambling risk status, and differences between groups were identified using Chi-square tests and ANOVAs. Thematic analysis was used to interpret qualitative data.

**Results:**

Almost two thirds (*n* = 324, 63.7%) of women had engaged with one of the four products in the previous 12 months. Compared to other age groups, younger women aged 16–34 years exhibited a higher proportion of problem gambling, gambled more frequently, and across more products. While EGMs were the product gambled on most frequently by women overall, younger women were significantly more likely to bet on sports and gamble at casinos relative to older women. Qualitative data indicated that younger women engaged with gambling products as part of a “night out”, “with friends”, due to their “ease of access” and perceived “chance of winning big”*.* There were significant differences in the perceptions of the harms associated with horse and sports betting according to age and gambling risk status, with younger women and gamblers perceiving these products as less harmful.

**Conclusions:**

This study highlights that there are clear differences in the gambling behaviour, product preferences, and perceptions of product harms between subgroups of women. A gendered approach will enable public health researchers and policymakers to ensure that the unique factors associated with women’s gambling are taken into consideration in a comprehensive public health approach to reducing and preventing gambling harm.

## Background

Historically, research has focused on men’s gambling attitudes and behaviour, with some researchers recognising a clear male bias in gambling research [1]. This is because men traditionally have significantly higher participation rates and harms from gambling as compared to women [2–4]. However, some research into women’s gambling participation rates demonstrates that participation in gambling is roughly similar for men and women [3–5]. For example, a 2011 New South Wales (NSW) prevalence study showed that 64% of women gambled at least once in that year, compared to 66% of men [4]. In New Zealand, 80.3% of women and 80.4% of men had gambled in the past year [3]. The most recent prevalence data from the UK indicated 44% of women and 53% of men had participated in some form of gambling in the past 4 weeks [5]. Research has also demonstrated that women who gamble frequently are highly susceptible to developing gambling problems [6], with women having a faster progression from initiation of gambling to the development of problems than men [7–9]. Prevalence studies have also indicated a significant increase in the number of women scoring at ‘low risk’ levels of gambling harm [2, 4]. For example, the Victorian Prevalence Study showed a 5.55% increase in females classified as low-risk gamblers from 4.44% in 2008 to 9.99% in 2014 [2].

Comprehensive approaches in other areas of public health, such as tobacco, have highlighted the importance of a gendered approach as part of comprehensive strategies to preventing and reducing the harms associated with smoking [10]. Researchers have argued for the "critical need to bring gender into mainstream tobacco control policies" (p. 891) [11], highlighting the range of gender specific factors that influenced women’s smoking behaviour, and the smoking-related harms experienced by women [12–15]. Researchers have also considered the range of socio-cultural, environmental, and industry tactics (such as the targeted marketing of cigarettes to women) that shaped women’s attitudes and beliefs about smoking, including their brand and product preferences [16]. This approach enabled the identification of the strategic targeting of women by tobacco companies [16–19], as well as understanding how women responded to a range of tobacco control policies and initiatives [13].

Despite the historical template provided by tobacco, no research or policy to our knowledge has specifically taken a gendered approach to reducing and preventing gambling harm. Furthermore, very few studies have specifically sought to understand the range of socio-cultural, environmental, and industry factors that may influence women’s gambling [1]. This is concerning as gambling is associated with entrenching health inequalities and social harms, particularly in areas of social deprivation [20–22]. Research which specifically focuses on women’s gambling is concentrated around three themes.

First are studies exploring the range of socio-cultural factors that may influence individual gambling behaviour. Loneliness and boredom are two factors that have been linked with gambling harm in women [23–25]. For example, studies indicate that gambling may be an ‘escape’ for women experiencing loneliness and mental health stress [26, 27] and that women who gamble because of loneliness are also more likely to experience gambling harm [24, 28]. Research suggests that women may use gambling as a way of coping with anxiety and tensions from social, household, or workplace demands, and to alleviate feelings of loneliness and depression [26, 29]. Women may also gamble for social reasons [30]. For example, an Australian study showed that women who experienced significant harm from gambling were motivated to gamble in order to be socially engaged [31]. Researchers have also highlighted the role of venue workers in providing women who attend gambling venues with a source of social connectedness [32], particularly during significant life events [31]. Research has also suggested that women’s gambling participation may be increasing due to it becoming more socially acceptable for women to attend gambling environments [25, 31]. For example, some studies have suggested that women see community clubs that contain gambling products as ‘safe environments’ in which they can meet up with friends [31, 33], but which ultimately may provide a pathway to engaging in harmful forms of gambling such as electronic gambling machines (EGMs).

Second, and perhaps less well understood, are differences between subgroups of women. For example, studies suggest that older women may be particularly vulnerable to gambling harm because of their increased social isolation, loneliness, lack of leisure alternatives, and physical health problems [34, 35]. Studies suggest that women’s gambling behaviour may increase as women get older as a way to occupy their time after retirement, and after their children leave home [36, 37]. Research shows that on average, older women spend more money on gambling products than younger women [38]. This is of particular concern given increased expenditures on gambling activities among older adults is associated with increased likelihood of experiencing gambling problems [39]. Other studies suggest that there is an increase in younger women’s participation in gambling due to the social acceptance and normalisation of newer forms of gambling [1, 23]. For example, newer online forms of gambling may create a unique (and often blurred) dimension to the previous public/private spaces associated with female gambling, with women engaging in gambling at a much younger age (including adolescents) [1].

Third is the impact of gambling industry strategies on behaviour and product preferences. Research suggests that there may be differences in product choices between different subgroups of women [1]. For example, while researchers have indicated that women prefer chance-based forms of gambling (such as lotteries, bingo, and EGMs) [25], there is some evidence that younger women’s product preferences may be changing towards skill-based forms of gambling such as wagering on horses [1, 2, 40]. There may be a number of reasons for these changes, including the development of new technologies which have led to increased and more accessible opportunities to gamble [1] and which may appeal to younger women [41]. This also includes specific marketing strategies used by the gambling industry that may appeal to women [42–44], which have been shown to influence women’s engagement with gambling [9, 23, 45]. For example, some experts suggest that betting marketing is being transformed to attract women through pink colour schemes and advertising campaigns that use language aimed to appeal to women [44], and through gambling companies' use of glamour and female celebrities to promote products [42].

This study aimed to contribute to the above research by understanding the range of factors that may influence women’s gambling behaviour, product preferences, and perceptions of product harm, and in particular, how these vary according to subgroups of women. This study was guided by four research questions:

1. How does gambling frequency vary by age and gambling risk status?
2. How do forms of gambling that women are participating in vary by age and gambling risk status?
3. Do women’s reasons for preferring some forms of gambling vary by age and gambling risk status?
4. To what extent do women’s perceptions of harm associated with gambling products vary by age and gambling risk status?

## Methods

### Data collection

The data presented in this paper was collected as part of a broader online panel study of men and women, which aimed to understand community attitudes towards gambling [46]. Approval for the research was obtained from the University Human Research Ethics Committee.

The questionnaire was programmed and administered using Qualtrics survey software. Data were collected between March and May 2017 from a convenience sample of 1000 Australians aged 16 years and older who lived in NSW and Victoria. This paper focuses specifically on the data provided by female participants (*n* = 509). A total of 2750 women accessed the survey. Of these, 46 women were excluded and replaced because of missing or unreliable data (for example illegible qualitative responses). Most women were screened out of the survey due to age quotas being filled, not consenting to participate in the study, or they did not complete each question in the survey. The decision to make women the sole focus of this study, rather than compare them to men as prior studies have typically done [26, 47–49], was made for two main reasons. First, we would argue that while studies that compare men and women are important, they may unintentionally downplay the harms experienced by women because of males’ propensity for greater risk taking and subsequent experiences of gambling harm. Second, considering women as one homogenous group can mask differences between subgroups of women that are important in understanding and explaining gambling attitudes and behaviour. It is therefore important to present standalone investigations which exclusively examine and report the evidence relating to women and different subgroups of women. This research aimed to extend existing work by exploring where there may be points of similarity and difference between different groups of women according to their age and gambling behaviour. Comparisons by age was chosen to be a focus for this study based on existing literature which suggests that there may be differences in product preferences between younger and older women, with younger women shifting from chance-based to skill-based products [1].

### Recruitment and sample

Convenience sampling techniques were used to recruit a sample that was representative (by age and state) of women aged 16 years and over living in NSW and Victoria [50, 51]. Although they are unable to legally gamble, 16- and 17-year-olds were included in the sample as previous research has shown that newer forms of gambling may appeal to younger women [41]. Participants were recruited through an online research panel company, which invited panel members to participate according to the age and sex quotas specified by the research team. Details of the study were sent to eligible panel members who voluntarily opted into the study. Participants registered with the online panel company receive points for completing surveys which can be redeemed for various products. The Plain Language Statement which accompanied the survey stated that participation was voluntary, prior to consenting to participate. While previous gambling participation was not necessary for eligibility, online panel studies tend to recruit more people who engage in gambling than other survey methods, possibly due to their online, anonymous, and confidential nature [25, 52, 53]. Therefore, gamblers and those who experience harm from gambling may have been overrepresented in this sample and may not be representative of the general female population in Australia.

### Measures

#### Socio-demographic characteristics

Data were collected on socio-demographic indicators (sex, postcode, education, and employment status), with postcodes used to determine Socio-Economic Indexes for Areas (SEIFA) status (a measure of socio-economic advantage and disadvantage) [54, 55]. Participants were also asked to type their age. The sample was split into three groups for the purpose of conducting comparisons: younger women (aged up to 34 years), middle-aged women (35–54), and older women (aged 55 years and over). There was not a large enough sample size to constitute a separate group consisting of 16 and 17 year-olds, and therefore these were classed in the younger women age group.

#### Problem gambling

The Problem Gambling Severity Index (PGSI) was used to measure problem gambling severity [56] and was administered to all participants. This index comprises nine items which ask questions about behaviours known to characterise problematic gambling, for example “When you think of the past 12 months, have you bet more than you could really afford to lose?” and “Have you felt guilty about the way you gamble or what happens when you gamble?” Participants then selected an answer from four options which were scored as ‘Never’ = 0, ‘Sometimes’ = 1, ‘Most of the time’ = 2, and ‘All of the time’ = 3. Scores of gambling risk status were summed, and individuals were grouped according to one of four groups: non-problem gambling (score 0), low-risk gambling (1–2), moderate-risk gambling (3–7), or problem gambling (8–27) [56]. Participants who scored 0 on the PGSI and indicated that they had not used any gambling products in the past 12 months were further classified as non-gamblers.

#### Gambling behaviour, preferences, and perceptions

Measures of gambling behaviour, preferences, and perceptions were specifically developed for this study using the C-OAR-SE method for scale development in the social sciences [57]. Using the C-OAR-SE method, the construct to be measured is first conceptually defined in terms of three elements: the object to be rated, the attribute on which it is rated, and the entity who will do the rating. Once defined, the construct elements are classified and the appropriate measure is developed.

#### Frequency of gambling and product use

Participants indicated how frequently they had participated in four types of harmful gambling activities in the past 12 months—casino gambling, EGMs, horse betting, and sports betting. Answers were indicated on a 5-point scale labelled ‘Never’ = 0, ‘Less than once a month’ = 1, ‘1-3 times per month’ = 2, ‘Weekly’ = 3, and ‘More than once a week’ = 4. Participants who had not taken part in any of the four gambling activities in the past 12 months were excluded from the analysis for research questions 1–3 which related specifically to gambling behaviour. Those who indicated that they had gambled previously on at least one of the four gambling products of interest were given a score calculated by summing the frequency scores (from 0 to 4) for the four gambling products, resulting in a score for each individual ranging from one (indicating gambling on one product less than once a month) to 16 (indicating gambling on all four products more than once a week). Those participants who were excluded for research questions 1–3 were added back in to the analysis for research question 4 which related to perceptions of gambling harm.

#### Gambling product preferences

To measure product preferences, individuals who did gamble were asked to think about the type of gambling they engaged in most and indicate why they preferred this type of gambling. Qualitative answers could be typed in an open-text field. This question was only asked to participants living in NSW.

#### Perceptions of harm

Participants were asked how harmful they thought each of the four gambling products (casino gambling, EGMs, horse betting, and sports betting) were, and could indicate their answer by sliding a marker to the appropriate point on a horizontal scale. The scale was labelled ‘Not at all harmful’ = 0 on the far left and ‘Extremely harmful’ = 100 on the far right. The point where the participant placed the maker was given the appropriate score between 0 and 100 to indicate the perceived level of harm for that product.

### Data analysis

Quantitative data were analysed using IBM Statistical Program for Social Sciences software, with descriptive statistics used to describe the total sample. Significant differences between groups were identified using Chi-square tests for categorical variables (research questions 1 and 2), and analysis of variance (ANOVAs) was performed to compare participants’ perceived level of harm for each product (research question 4). Significant Chi-square tests were followed by logistic regression models which produced point estimates for the odds ratio (OR) to examine the nature and size of such effects. Significant ANOVAs were followed by Tukey HSD tests which were also run to determine how mean harm scores differed between specific age groups and gambling risk status groups. The criterion of .05 was used in significance testing for these variables.

To address research question 3, qualitative responses were clustered into themes relating to reasons for gambling on different products. These themes were then compared within and across the sample.

## Results

### Sample description

Sample characteristics are shown in Table 1. The sample was grouped into three categories according to age (16–34, 35–54, and 55 and over), each consisting of approximately a third of participants. The sample was highly educated with 39.9% (*n* = 203) of women having completed a bachelor’s degree, graduate diploma/certificate, or post graduate qualification, and most participants (*n* = 424, 83.3%) were living in medium to high areas of socio-economic advantage (SEIFA scores of four and above). One third of participants reported some level of gambling harm (PGSI of 1 or more) (*n* = 170, 33.4%), with over one in five categorised as either a moderate risk or problem gambler (PGSI score 3 or above, *n* = 110, 21.6%).

**Table 1:** Socio-demographic and gambling behaviour of women (*n* = 509)

Characteristic	*n*	Percentage of sample
Age		
16–34 (younger)	171	33.6
35–54 (middle-aged)	166	32.6
55+ (older)	172	33.8
State of residence		
NSW	254	49.9
VIC	255	50.1
Education		
Year 12 or below	166	32.6
Cert I, II, III, IV	68	13.4
Diploma/advanced	72	14.1
Bachelor’s degree	135	26.6
Graduate diploma/certificate	22	4.3
Post graduate	46	9.0
Employment		
Working full-time	149	29.3
Working part-time or casually	132	25.9
Unemployed but looking for work	19	3.7
Homemaker	54	10.6
Retired	108	21.2
Full-time student	42	8.3
Other	5	1.0
Socio-economic area (SEIFA status)*		
Low (1–3)	83	16.3
Middle (4–7)	209	41.1
High (8–10)	215	42.2
Gambling risk status		
Non-gambling	104	20.4
Non-problem gambling	235	46.2
Low-risk gambling	60	11.8
Moderate-risk gambling	48	9.4
Problem gambling	62	12.2

*Note: two postcodes did not have SEIFA scores assigned to them so were excluded from this table

Given that the focus of this study was on different groups of women based on age and problem gambling risk status, cross tabulations were run to identify the number of young, middle-aged, and older women with different levels of problem gambling behaviour (Table 2). Age group was significantly associated with gambling risk status [*χ*^2^(8, *N* = 509) = 54.90, *p* < .001]. Younger women aged 16–34 indicated experiencing the most gambling harm, with just under half of this age group scoring as either low-risk, moderate-risk, or problem gamblers (*n* = 77, 45.0%). Additionally, younger women were also the age group with the highest proportion of problem gamblers (*n* = 40, 23.4%). This was compared to 10.2% (*n* = 17) of 35 to 54 year-olds and only 2.9% (*n* = 5) of women aged 55 and over classified as problem gamblers. The OR demonstrated that younger women aged 16–34 were 2.68 times more likely than middle-aged women aged 35–54 and 10.20 times more likely than older women aged 55 and over to be classified as a problem gambler. Just over 60% (*n* = 104) of women aged 55 and over were classified as non-problem gamblers, indicating that these women gambled but not at harmful levels. According to the OR, older women 55 and over in this sample were 1.77 times more likely to be classified as non-problem gamblers than middle-aged women and 3.31 times more likely than younger women.

**Table 2:** Cross tabulation of age of women by gambling risk status

Gambling risk status	Age						Significance
	16–34 (*n* = 171)		35–54 (*n* = 166)		55+ (*n* = 172)		
	*n*	%	*n*	%	*n*	%	
Non-gambler	40	23.4	27	16.3	37	21.5	*χ*^2^ = 54.90, *p* < .001
Non-problem	54	31.6	77	46.4	104	60.5	
Low risk	18	10.5	26	15.7	16	9.3	
Moderate risk	19	11.1	19	11.4	10	5.8	
Problem	40	23.4	17	10.2	5	2.9	

Note: *n* = actual number of participants and % = column percentages

### Gambling frequency

Based on the distribution of frequency scores (range 1–16), the sample was split into three approximately even groups based on relative frequency of gambling on the four products (*n* = 324). Those with a score of 1 were the low-frequency group, scores of 2 and 3 were the medium-frequency group, and scores of 4 or more were the high-frequency group. Results of cross-tabulations between (1) frequency groups and age groups and (2) frequency groups and gambling risk status groups are shown in Table 3. Significant differences were found between age groups and the frequency of gambling [*χ*^2^(4, *N* = 324) = 14.03, *p* = .007], with 45.0% (*n* = 49) of younger women gamblers aged 16–34 in the high-frequency group and only 22.2% (*n* = 22) in the low-frequency group. Conversely, only 22.0% of older women aged 55 or over were in the high-frequency group, with almost double this number (*n* = 39, 39.4%) in the low-frequency group. The OR demonstrated that younger women were 1.97 times more likely than middle-aged and older women to gamble at high frequencies. Additionally, older women were 1.78 times more likely than middle-aged women and 2.30 times more likely than younger women to gamble at low frequencies.

**Table 3:** Frequency of women’s gambling by age and gambling risk status (*n* = 324)

	Frequency						Significance
	Low		Medium		High		
	*n* = 94		*n* = 118		*n* = 112		
Age	*n*	%	*n*	%	*n*	%	
16–34	24	22.0	36	33.0	49	45.0	*χ*^2^ = 14.03, *p* = .007
35–54	31	26.7	44	37.9	41	35.3	
55+	39	39.4	38	38.4	22	22.2	
Gambling status							
Non-problem	74	44.3	69	41.3	24	14.4	*χ*^2^ = 114.18, *p* < .001
Low risk	12	21.1	28	49.1	17	29.8	
Moderate risk	6	14.6	15	36.6	20	48.8	
Problem	2	3.4	6	10.2	51	86.4	

Note: *n* = actual number of participants and % = row percentages

A significant association was also found between the gambling risk status of women and how frequently they gambled on the four products [*χ*^2^(6, *N* = 324) = 114.18, *p* < .001]. The majority (*n* = 51, 86.4%) of problem gamblers gambled at ‘high’ frequencies, with only 3.4% (*n* = 2) of problem gamblers gambling at low frequencies. According to the OR, problem gamblers were 37.98 times more likely than non-problem gamblers, 15.00 times more likely than low-risk gamblers and 6.69 times more likely than moderate-risk gamblers to have gambled at ‘high’ frequencies. In comparison, non-problem gamblers were 2.98 times more likely than low-risk gamblers, 4.64 times more likely than moderate-risk gamblers, and 22.68 times more likely than problem gamblers to gamble at ‘low’ frequencies.

### Gambling product use

Results of cross-tabulations between gambling product use and (1) age groups and (2) gambling risk status groups are shown in Table 4.

**Table 4:** Women’s gambling product use by age and gambling risk status (*n* = 324)

	Gambling product							
	EGMs		Horse betting		Casino		Sports betting	
	*n* = 250, 77.2%		*n* = 206, 63.6%		*n* = 166, 51.2%		*n* = 122, 37.7%	
Age	*n*	%	*n*	%	*n*	%	*n*	%
16–34	86	34.4	69	33.5	73	44.0	62	50.8
35–54	92	36.8	77	37.4	57	34.3	44	36.1
55+	72	28.8	60	29.1	36	21.7	16	13.1
Significance	*χ*^2^ = 1.60, *p* = .450		*χ*^2^ = .77, *p* = .679		*χ*^2^ = 19.77, *p* < .001		*χ*^2^ = 36.65, *p* < .001	
Gambling status	*n*	%	*n*	%	*n*	%	*n*	%
Non-problem	117	46.8	100	48.5	63	38.0	30	24.6
Low risk	44	17.6	34	16.5	25	15.1	26	21.3
Moderate risk	36	14.4	24	11.7	25	15.1	18	14.8
Problem	53	21.2	48	23.3	53	31.9	48	39.3
Significance	*χ*^2^ = 12.79, *p* = .005		*χ*^2^ = 9.87, *p* = .020		*χ*^2^ = 50.18, *p* < .001		*χ*^2^ = 77.80, *p* < .001	

Note: *n* = actual number of participants and % = column percentages

Based on their responses to questions about product use, women were given binary scores to indicate whether they had used each product in the past 12 months or not. No significant differences were found between age groups in the use of EGMs and horse betting. However, differences were found between age groups and women gambling at casinos [*χ*^2^(2, *N* = 166) = 19.77, *p* < .001] and age groups and women betting on sports [*χ*^2^(2, *N* = 122) = 36.65, *p* < .001]. Of those who bet on sport, over half were aged 16–34 (*n* = 62, 50.8%). In comparison, 36.1% (*n* = 44) of 35 to 54 year-olds, and 13.1% (*n* = 16) of women aged 55 and over bet on sports. According to the OR, 16 to 34 year-old women were 2.15 times more likely than middle-aged women and 6.84 times more likely than older women to have previously bet on sports (as opposed to never having bet on sports). Similarly, women who gambled at the casino were most likely to be aged 16–34 (*n* = 73, 44.0%), and least likely to be aged 55 and over (*n* = 36, 21.7%). Finally, 16 to 34 year-olds were 2.10 times more likely than middle-aged women and 3.55 times more likely than older women to have gambled at the casino.

Differences were also found between different gambling risk status groups. Those participating in sports betting were significantly more likely to be women classified as either low-risk, moderate-risk, or problem gamblers [*χ*^2^(3, *N* = 122) = 77.80, *p* < .001]. Over 75% (*n* = 92) of women who participated in sports betting were classified as either low-risk, moderate-risk, or problem gamblers, which included over a third (*n* = 48, 39.3%) of sports betters classified as problem gamblers. The OR demonstrated that individuals classified as either a low-risk, moderate-risk, or problem gambler were 6.46 times more likely to have bet on sports in the previous year than non-problem gamblers.

Cross-tabulations were conducted to assess whether age and gambling risk status were associated with the number of different gambling products women engaged in (Table 5). A significant association was found between the age of participants and the number of products they participated in [*χ*^2^(6, *N* = 324) = 29.74, *p* < .001]. Younger women (aged 16–34) were the group most likely to gamble on all four gambling products (casinos, EGMs, horse betting, and sports betting), with over one third (*n* = 41, 37.6%) indicating that this was the case. The OR demonstrated that younger women were 2.09 times more likely than middle-aged women and 6.03 times more likely than older women to use all four products. Conversely, older women (aged 55 and over) were 3.01 times more likely than younger women and 2.27 times more likely than middle-aged women to use just one type of gambling product (*n* = 48, 48.5%).

**Table 5:** Cross tabulation of age by number of gambling products used in the last 12 months (*n* = 324)

Gambling product	Age								Significance
	16–34		35–54			55+			
	*n*	%	*n*		%	*n*		%	*χ*^2^ = 29.74, *p* < .001
1 product used	26	23.9	34		29.3	48		48.5	
2 products used	26	23.9	36		31.0	26		26.3	
3 products used	16	14.7	20		17.2	16		16.2	
4 products used	41	37.6	26		22.4	9		9.1	
Total	109	100.0	116		100.0	99		100.0	
Gambling product	Gambling risk status								Significance
	Non-problem		Low risk		Moderate risk		Problem		
	*n*	%	*n*	%	*n*	%	*n*	%	*χ*^2^ = 99.97, *p* < .001
1 product used	80	47.9	17	29.8	8	19.5	3	5.1	
2 products used	45	26.9	20	35.1	14	34.1	9	15.3	
3 products used	28	16.8	8	14.0	9	22.0	7	11.9	
4 products used	14	8.4	12	21.1	10	24.4	40	67.8	
Total	167	100	57	100	41	100	59	100	

Note: *n* = actual number of participants and % = column percentages

A significant association was also found between women of different gambling risk status and the number of products used [*χ*^2^(9, *N* = 324) = 99.97, *p* < .001]. According to the OR problem gamblers were 23.01 times more likely than non-problem gamblers, 7.90 times more likely than low risk gamblers and 6.53 times more likely than moderate risk gamblers to gamble on all four products.

### Gambling product preferences

Qualitative data revealed a range of reasons relating to women’s preferences for particular gambling products, with some evidence to suggest that product preferences varied by age group.

#### EGMs

There were differences in qualitative responses for EGM preferences between subgroups of women. Women aged over 35 stated that EGMs were the form of gambling that they enjoyed the most because they were “entertaining”, “enjoyable”, “exciting”, or “fun”. Some also commented that they preferred EGMs because they were easily accessible, easy to gamble on, and “convenient”. However, a few women distanced themselves from EGMs as a form of gambling, with one 65-year-old woman stating that she used EGMs “more for fun than actually gambling”. One woman perceived that there was more transparency around gambling losses on EGMs, stating that EGM gambling was entertaining and that “I know exactly how much I will lose, if I lose”*.* Younger women who stated that EGMs were their most preferred form of gambling stated that it was a form of gambling they could participate in as a social activity. For example, the following 25-year-old stated: “I’ll put a couple dollars in the pokies while drinking with friends”; while a 19-year-old stated “I don't really gamble, but the pokies can be a bit of fun when you're out with friends”.

#### Horse betting

Women, who described gambling on horses as their most preferred form of gambling, described that they enjoyed horse betting and that it was “cheap”, “convenient”, and “fun”. Some described the excitement associated with horse races, including the “thrill of horses racing”. Younger women (aged 16–34) regularly commented that horse racing was the form of gambling where you had more chance of winning “big” money. However, some of these women were also experiencing gambling harm. For example, a 21-year-old, who was experiencing moderate risk levels of gambling, believed that there was “more of a possibility to win” on horse betting. Women aged over 55 described horse betting as their preferred form of gambling because of its link with iconic cultural events. For example, women often described the Melbourne Cup (a horse race associated with a public holiday in the Australian state of Victoria), as the only reason they chose to bet on horses and viewed this event as more of a social event than a “gambling day”.

#### Casino gambling

Very few women chose casino gambling as their most preferred form of gambling, with the majority of women who chose this under the age of 35. Younger women described the broader entertainment associated with the casino, and casino gambling as part of a “night out” and something that was done “for fun”.

#### Sports betting

For those women who preferred sports betting, the predominant theme was associated with how easy it was to access sports betting products. Women who participated in sports betting on a weekly basis stated that they preferred sports betting because they believed it was a form of gambling where people could win a lot of money. Two women who were problem gamblers stated that sports betting was their most preferred form of gambling because it was “fun”. There were different reasons for sports betting as a preferred product across age groups. Women over 55 described sports betting as “low cost”, while middle-aged women (aged 35–54) often described sports betting as a low-risk form of gambling. Some of these women were also experiencing low-risk levels of gambling harm. For example, the following 37-year-old low-risk gambler stated that she preferred sports betting because “it is easy and the risk of losing money is low”*.* One 16-year-old who screened as a problem gambler stated that she preferred sports betting because of how easy it was to access.

### Perceptions of harm

A one-way independent measure, ANOVA, was used to investigate participants’ perceived level of product harm between women of different age groups and gambling risk status groups. Inspection of Levene’s statistic found that the assumption of homogeneity of variance was met for all age groups and gambling risk groups except for gambling risk groups when measuring perceived level of sports betting harm. To compensate for the violation of the homogeneity of variance assumption, the Brown-Forsythe ANOVA result was used. The mean harm scores of each gambling product of interest by age and gambling risk status are presented in Table 6.

**Table 6:** Mean harm scores for gambling products by age and gambling risk status

	Gambling product (mean score)			
	Casino	EGMs	Horse betting	Sports betting
	(*M* = 78.12)	(*M =* 76.00*)*	(*M* = 70.31)	(*M* = 69.75)
Age				
16–34	74.81	73.11	66.92	64.92
35–54	79.27	78.27	70.64	69.78
55+	80.31	76.70	73.35	74.52
Gambling status				
Non-gambler	81.70	78.88	76.35	75.17
Non-problem	79.34	77.47	72.11	72.02
Low risk	72.38	70.48	62.87	62.90
Moderate risk	76.56	74.23	63.75	63.52
Problem	74.26	72.34	65.60	63.50

Overall, women in this sample perceived casino gambling (*M* = 78.12) and EGMs (*M* = 76.00) as the most harmful gambling products. However, there were no significant differences in perception of harm for these two products according to age or gambling risk status. There were however significant associations between age and gambling risk status with horse betting and sports betting.

#### Horse betting

Age had a significant effect on perception of harm towards horse betting [*F*(2, 506) = 3.09, *p* = .046]. The mean score given by younger women aged 16–34 years was significantly lower than the mean score given by older women aged 55 and over (*p* = .036).

Gambling risk status also had a significant effect on perception of harm towards horse betting groups [*F*(4, 504) = 5.01, *p* = .001], with non-gambling women considering horse betting to be significantly more harmful, when compared to low-risk gamblers (*p* = .005), moderate-risk gamblers (*p* = .021), and problem gamblers (*p* = .040).

#### Sports betting

Age had a significant effect on perception of harm towards sports betting [*F*(2, 506) = 6.57, *p* = .002]. The mean score given by younger women aged 16–34 was significantly lower than the mean score given by older women aged 55 and over (*p* = .001).

Gambling risk status also had a significant effect on perception of harm towards sports betting [*F*(4, 504) = 4.77, *p* = .001], with non-gambling women considering sports betting to be significantly more harmful than low-risk gamblers (*p* = .017), moderate-risk gamblers (*p* = .050), and problem gamblers (*p* = .025).

## Discussion

The study raises a number of points for discussion.

First, younger women have different risks of gambling harm compared to older women. Younger women were more likely to experience severe levels of gambling harm, with just under one quarter being classified as problem gamblers. This compares to the older age group which only had 3% classified as problem gamblers, and the middle-age group which had around 10% problem gamblers. Middle-aged and older women had the highest proportion of women who were not experiencing any harm from their gambling. This finding perhaps raises more questions than answers, including how different groups of women may conceptualise gambling harm and why this discrepancy occurs. This could be due to a buffer effect whereby middle-aged and older women are more financially stable, so their perceptions of losses (and harms) are underestimated. Alternatively, there could be other factors that influence how different women conceptualise the risks and benefits of gambling. For example, Thomas and Lewis (2012) found older women had a lower perception of harm associated with their own gambling in EGM venues because they felt that there was a trade-off between the non-gambling incentives (for example, cheap meals) and social benefits that they perceived were associated with venues (for example, social interaction and inclusion), and the money that they lost on EGMs [32]. Further research should seek to understand how women conceptualise gambling harm, and how this differs between subgroups of women. This type of research may enable the development of more robust measures of gambling harm that are specifically relevant to women’s experiences. Research will also need to adapt to the changing gambling landscape to incorporate all aspects of gambling including both venue based and online gambling.

Second, younger women gambled the most frequently compared to any other age group. This suggests that gambling may be becoming a more normal and regular part of young women’s lives than for previous generations. While accessibility and availability of products are recognised as influencing factors in gambling behaviour [25, 58], we would argue that there may also be a specific range of gambling industry strategies, such as targeted advertising, that influence the socio-cultural acceptance and normalisation of gambling for younger women. While some research has examined the factors that influence the normalisation of young men’s gambling [59, 60], there has been limited studies that have sought to understand the factors contributing to the normalisation and social acceptance of gambling in younger women. Such studies should seek to investigate the range of gambling and non-gambling activities that may encourage women’s engagement with a range of gambling environments, including clubs, pubs, and casinos, and the impact this has on their gambling attitudes and behaviour. Further, while our sample of underage gamblers was too small to yield significant results, these preliminary findings show that women aged 16 and 17 report gambling and are at risk of experiencing harm.

Third, the extent of women’s gambling participation and their preferences for products varied across age group, with younger women significantly more likely to gamble on multiple products and older women most likely to gamble on just one product. The findings from this study are in contrast with other studies which have shown that women gamble on relatively few gambling products [47, 61]. These results suggest that rather than *shifting* from chance to skill-based forms of gambling, younger women are *diversifying* their product engagement to gamble on multiple products, with horse and sports betting added to existing forms of chance-based gambling, such as EGMs. One surprising finding was the number of young women who had engaged in sports betting and the number of these women who were experiencing gambling harm. There are a number of potential explanations for this. First is the increased exposure to promotions for online gambling. While young men are currently the key target market for online bookmakers, women, and particularly those who watch sport, are also exposed to the advertising for these products. We would hypothesise that exposure to these advertisements may have a normalising impact on women’s attitudes towards newer forms of gambling. Second, we should not assume that online bookmakers are only interested in targeting men. Research investigating the strategies used by the gambling industry to target women should include social media platforms, ‘direct to consumer’ forms of marketing, including emails, SMS messages, and phone calls that women may receive after signing up for accounts. There is also some evidence that corporate bookmakers are starting to develop advertisements which may appeal to women, and which feature women in lead roles in these promotions [62]. Third, we should not make the assumption that there is a clear gender divide with gambling in peer groups—that men only gamble with other men and women only gamble with other women. Finally, younger women more often described gambling as a social activity and a form of entertainment as compared to older women, suggesting that there may also be a degree of socio-cultural acceptance associated with gambling in younger women.

Finally, while women in this study perceived all gambling products as harmful, younger women and gamblers (particularly in low and moderate-risk groups) had a lower perception of the harms associated with some gambling products (horse and sports betting), as compared to older women and non-gamblers. This raises questions about why these groups of women are less likely to perceive the harms associated with these products, and what strategies could address this. As non-gamblers perceived gambling products as most harmful, it is hypothesised that perceptions of harm influences engagement with gambling products. Therefore, one explanation of the results is that there may be an association between younger women’s perceptions of product harm and their frequent use of gambling products. Research from other areas of public health has demonstrated that, in general, the more harmful an individual’s perception of a product, and the more they perceive they are at personal risk of experiencing harm, the less likely they are to initiate use of the product [63]. While mean harm perception scores indicated a level of awareness of the harms associated with gambling products, more needs to be done to protect those most at risk. One strategy may therefore be to develop campaigns which highlight the harms associated with particular types of gambling products. While the vast majority of gambling harm minimisation campaigns are related to ‘responsible gambling’ behaviour and help seeking [64], there is strong support from the literature relating to other health behaviour and from communities for education relating to product harm [10, 13, 65]. It may also be appropriate to consider ‘lay epidemiology’ approaches which entail paying more attention to individuals’ own experiences and perceptions [66–68].

### Limitations

This sample was skewed towards women who had higher levels of education and lived in affluent areas, with most having completed at least the final year of high school and were from medium to high areas of socio-economic advantage (SEIFA scores above 4). Data were only collected from two states in Australia and may not be generalisable to the entire Australian population. Online panel studies tend to recruit more people who engage in gambling activities, and therefore the sample contains an overrepresentation of women experiencing gambling problems as compared to community-based prevalence studies. However, online panels may reach a population of online gamblers that telephone surveys do not and are particularly important for understanding the gambling behaviour of younger adults [52]. This study involved secondary analyses of data from a larger study and was thus associated with a fixed sample size. Post hoc power analyses indicated that this sample was associated with 80% power for detecting a proportion difference between two groups of around 7% (assuming equally sized groups and a 5% proportion in the referent condition) and was not adequately powered for detecting smaller effects. Further, assumptions of normality were not met for dependent variables. However, supplementary analyses using non-parametric tests were run and these support identical conclusions. Finally, the study only analysed women’s engagement with four gambling products and does not take into consideration other popular gambling products, such as lotteries, bingo, and Keno.

## Conclusion

The study identified differences in the gambling behaviour, product choices, and perceptions of gambling harm between different subgroups of women, particularly between women of different ages and gambling risk status. These findings highlight the importance of a gendered approach to gambling research, which would consider the different risks and needs of women, and the development of specific policies and initiatives aimed at reducing gambling harm in different groups of women as part of a comprehensive approach.

## Abbreviations

ANOVA: Analyses of variance
EGM: Electronic gambling machine
NSW: New South Wales
OR: Odds Ratio
PGSI: Problem Gambling Severity Index
SEIFA: Socio-Economic Indexes for Areas
